# Perceptions of Prehospital Care for Patients With Limited English Proficiency Among Emergency Medical Technicians and Paramedics

**DOI:** 10.1001/jamanetworkopen.2022.53364

**Published:** 2023-01-27

**Authors:** Kathryn M. Stadeli, Dylan Sonett, Kelsey M. Conrick, Megan Moore, Matthew Riesenberg, Eileen M. Bulger, Hendrika Meischke, Monica S. Vavilala

**Affiliations:** 1 Department of Surgery, University of California, Davis, Sacramento; 2 Department of Surgery, University of Washington, Seattle; 3 Harborview Injury Prevention and Research Center, University of Washington, Seattle; 4 Physical Sciences Division, University of Washington Bothell, Seattle; 5 School of Social Work, University of Washington, Seattle; 6 King County Medic One, King County Emergency Medical Services, Seattle, Washington; 7 Department of Health Systems and Population Health, University of Washington, Seattle; 8 Department of Anesthesiology and Pain Medicine, University of Washington, Seattle

## Abstract

**Importance:**

Patients with limited English proficiency (LEP) experience disparities in prehospital care. On-scene interactions between patients with LEP and emergency medical services (EMS) providers (ie, firefighters/emergency medical technicians [EMTs] and paramedics) are critical to high-quality care and have been minimally explored.

**Objective:**

To identify EMS-perceived barriers and facilitators to providing high-quality prehospital care for patients with LEP.

**Design, Setting, and Participants:**

In this qualitative study, semi-structured focus groups were conducted with firefighters/EMTs and paramedics with all levels of experience from urban areas with a high proportion of residents with LEP from July to September 2018. Data were analyzed from July 2018 to May 2019.

**Exposures:**

Providing prehospital care for patients with LEP.

**Main Outcomes and Measures:**

The main outcomes were barriers and facilitators to prehospital care for patients with LEP, assessed using thematic analysis. Four domains of interest were examined: (1) overall impressions of interactions with patients with LEP, (2) barriers and facilitators to communication, (3) barriers and facilitators to providing care, and (4) ideas for improving prehospital care for patients with LEP.

**Results:**

Thirty-nine EMS providers participated in 8 focus groups: 26 firefighters/EMTs (66%) and 13 paramedics (33%). The median age of participants was 46 years (range, 23-63 years), and 35 (90%) were male. Participants described barriers to optimal care as ineffective interpretation, cultural differences, high-stress scenarios (eg, violent events), unclear acuity of patient’s condition, provider bias, and distrust of EMS. Perceived facilitators to optimal care included using an on-scene interpreter, high-acuity disease, relying on objective clinical findings, building trust and rapport, and conservative decision-making regarding treatment and transport. Providers reported transporting most patients with LEP to hospitals regardless of illness severity due to concern for miscommunication and unrecognized problems. Better speed and technology for interpretation, education for communities and EMS providers, and community-EMS interactions outside emergencies were cited as potential strategies for improvement.

**Conclusions and Relevance:**

In this study, EMS providers described many barriers to high-quality care during prehospital emergency response for patients with LEP yet were unaware that these barriers impacted quality of care. Barriers including ineffective interpretation, provider bias, distrust of EMS, and cultural differences may contribute to outcome disparities and overutilization of resources. Future work should focus on the development of targeted interventions to improve modifiable barriers to care, such as improving interpretation and cultural humility and increasing trust.

## Key Points

**Question:**

What factors do emergency medical services (EMS) providers (ie, firefighters/emergency medical technicians [EMTs] and paramedics) perceive as barriers to prehospital care for patients with limited English proficiency (LEP)?

**Findings:**

In this qualitative study with 39 EMS providers (26 firefighters/EMTs and 13 paramedics), ineffective language interpretation, cultural differences, and distrust were identified as important barriers to care for patients with LEP.

**Meaning:**

In this study, EMS providers described several barriers to providing high-quality prehospital care for patients with LEP that may contribute to disparities in outcomes.

## Teaser

This qualitative study assesses factors perceived by firefighters/emergency medical technicians (EMTs) and paramedics as barriers and facilitators to prehospital care for patients with limited English proficiency (LEP).

## Introduction

Patients with limited English proficiency (LEP) face barriers to involvement of and communication with emergency medical services (EMS) providers (firefighters/emergency medical technicians [EMTs] and paramedics) during prehospital emergencies that contribute to disparities in outcomes.^1,2,3,4,5,6,7^ While most available information on outcomes (such as time to dispatch and survival) is related to 911 dispatch and cardiac events,^1,4,8,9^ the inequities identified are relevant to other aspects of prehospital care, including accessing the EMS system, communication and interpersonal interactions on scene, provision of care, and medical decision-making.

Most prior prehospital studies focused specifically on language barriers, but other factors such as cultural differences, bias, and discrimination likely also impact care and outcomes for patients with LEP.^10,11,12,13^ From 2014 to 2018, approximately 8.5% of the US population spoke English less than “very well,”^14^ and 21.5% spoke a language other than English at home.^14,15^ In the few studies that have examined access to EMS from the perspective of individuals with LEP, some of the primary barriers included language barriers, financial concerns, fear and distrust of law enforcement and/or litigation, negative perceptions of EMS, immigration status, and confusion with the EMS system.^6,16,17^ There is limited information on prehospital care for patients with LEP from the perspective of EMS providers. Yet, there are important lessons to learn regarding the challenges faced by EMS providers, strategies used, and areas for targeted interventions to improve equity. We aimed to identify EMS-perceived factors impacting prehospital care for patients with LEP.

## Methods

### Study Design

In this qualitative study, we explored EMS provider experiences and perspectives on prehospital response for patients with LEP using phenomenological qualitative methods. We conducted focus groups to promote discussion among providers and to provide different views. The University of Washington Human Subjects Division Institutional Review Board determined the study to be exempt because of minimal risk to participants and deidentification of data. Written informed consent was obtained. The study adhered to the Standards for Reporting Qualitative Research (SRQR) reporting guideline.^18^

### Setting

In 2018 in King County, Washington, 10% of the residents had LEP and 28% spoke a language other than English at home.^14^ The most common non-English languages were Spanish, Chinese (all dialects), Vietnamese, East African languages (Somali and Amharic), and Korean.^15^ Regarding EMS, basic life support (firefighters/EMTs) is dispatched for all medical calls while advanced life support (2 paramedics) is dispatched for high-acuity patients.^19^ Basic life support can add or cancel advanced life support.

### Selection of Participants

Firefighter/EMTs and paramedics were purposively sampled from stations where responses for patients with LEP are common. Providers with all levels of EMS experience were included. Race and ethnicity were self-reported as American Indian/Alaska Native, Asian, Black/African American, Native Hawaiian/Pacific Islander, White, and other (not specified). Race, ethnicity, and language were reported to examine how EMS providers may or may not be representative of the communities they serve.

### Semi-Structured Focus Groups

Semi-structured focus groups were conducted from July to September 2018 at fire stations during EMS provider shifts. We conducted 2 focus groups with only firefighters/EMTs, 1 with only paramedics, and 5 with all provider types based on availability (range, 2-9 providers per group). The moderator guide (eAppendix in Supplement 1) addressed 4 domains of interest: (1) overall impressions of interactions with patients with LEP, (2) barriers and facilitators to communication, (3) barriers and facilitators to providing care, and (4) ideas for improving prehospital care for patients with LEP. All researchers approved the interview guide. A researcher trained in qualitative methods (K.M.C.) trained and supervised an EMT-trained research assistant (D.S.) to moderate focus groups. After obtaining informed consent, all focus groups were recorded, transcribed verbatim (D.S.), reviewed for accuracy (K.M.S.), and deidentified.

### Analysis

Two researchers (K.M.S., D.S.) analyzed data with Dedoose software (SocioCultural Research Consultants)^20^ using iterative inductive thematic analysis from July 2018 to May 2019. Analysis was reviewed in May 2022 with no major changes (K.M.S.). Researchers defined thematic saturation as no new themes arising in at least 2 focus groups. After 6 focus groups, a codebook was developed by consensus. All data were reanalyzed using consensus codes. Two additional focus groups were conducted and analyzed to confirm thematic saturation. Results are presented as barriers, facilitators, EMS provider–reported effects of LEP on their decision-making, and EMS provider recommendations for improvement.

## Results

### Participant Characteristics

Thirty-nine EMS providers participated in 8 focus groups: 26 firefighters/EMTs (66%) and 13 paramedics (33%) (Table 1). The median age of participants was 46 years (range, 23-63 years); 4 (10%) were female, and 35 (90%) were male. One participant (3%) identified as American Indian/Alaska Native; 1 (3%), Asian; 1 (3%), Black/African American; 36 (92%), White; and 2 (5%) other race and ethnicity. Median EMS experience was 18 years (range 0-43 years), and 6 participants (15%) spoke Spanish in addition to English (Table 1).

**Table 1.** EMS Participant Characteristics

Characteristic	Participants, No. (%)		
	Firefighters/EMTs (n = 26)	Paramedics (n = 13)	All (N = 39)
Sex			
Female	0	4 (31)	4 (10)
Male	26 (100)	9 (69)	35 (90)
Age, median (range), y	42 (28-63)	49 (23-62)	46 (23-63)
Time of EMS experience, median (range), y	14 (0-39)	25 (12-43)	18 (0-43)
Race and ethnicity			
American Indian/Alaska Native^a^	1 (4)	0	1 (3)
Asian^a^	1 (4)	0	1 (3)
Black/African American	1 (4)	0	1 (3)
Native Hawaiian/Pacific Islander	0	0	0
White	23 (88)	13 (100)	36 (92)
Other^b^	2 (8)	0	2 (5)
Languages spoken in addition to English			
Spanish	4 (15)	2 (15)	6 (15)
German	2 (8)	0	2 (5)
French	1 (4)	0	0

Abbreviations: EMS, emergency medical services; EMT, emergency medical technician.

^a^ Individuals who identified as American Indian/Alaska Native and Asian also identified as White.

^b^ Individuals who identified as other did not specify a racial or ethnic identity.

### Barriers to Optimal Prehospital Care

The most common barrier identified across focus groups was ineffective language interpretation (Table 2 and eTable 1 in Supplement 1). Providers consistently described telephonic interpreting as inefficient and used it as a last resort in cases in which detailed history was important to determine disposition. Another related, frequent barrier was unclear severity of patient condition (eg, abdominal pain of unknown etiology) because patients required a detailed history and nuanced examination for decision-making, which depended on the effectiveness of interpretation.

**Table 2.** Barriers to Optimal Prehospital Care

Barrier	Quote
Ineffective interpretation^a^^,^^b^	“…it depends on the interpreter you get, sometimes…sometimes you just get this long lag, and you’re really not sure, really have very little confidence…so it really kinda depends on who’s the other end of the line.”
Unclear acuity of patient’s condition^c^	“…presented as abdominal pain…vitals were completely fine, everything that we could check was fine…there was no ALS indicators from anything we could gather…he arrested on the ramp at [hospital], had a massive MI.…I literally did everything I could’ve done in that scenario…but, he couldn’t really tell me, he’s kinda grabbing his stomach.”
High-stress scenarios	
Violent event	“…we go to calls where there’s been some violence, and nobody’s communicating to us, because, either, they don’t, they don’t want the downstream effect by talking to us, or, they’re fearful of reprisal, or…I don’t know.”
Crowding of scene	“…like the car accident I had, they called up their friends before they called 911, and 30 of their friends showed up and started screaming at me in the middle of an intersection…her friend was just trying to translate what was wrong with her…I was glad she was there, but oh, everyone else…it’s the reverse of helpful.”
EMS authoritative attitude	“Some guys you work with try to be the very authoritative, and that never goes well. And somehow they think that big ‘You’re gonna answer my questions’ and it just, doesn’t go well.”
Cultural differences	“…can’t really separate the language, limited English, from the cultural aspect…like asking a woman to remove her hijab because she hit her head on the wall, and you need to be able to do, examine it, and you got a lot of family members around that maybe aren’t real comfortable with you doing that, or her removing it in front of you.”
Perception of delay in patients activating EMS^c^	“…I think your…Southeast Asia communities, [name of country], stuff like that, they tend to wait ‘til the last minute to call 911, or not even call it at all.”
Perception of increased illness severity^c^	“…the sickest babies that I usually get are from non-English speakers, …they’ve been sick, they’ve been septic, they didn’t realize that their infant was sick and by the time I get there it’s a critically, critically ill child, or it’s congenital defects or its issues from premature birth because they have had no health care.”
Distrust of EMS	
Negative prior experiences with EMS^a^	“Eastern bloc countries, when they called the fire department, the fire department robbed them. And so, there’s just that, mistrust of us from what they know from their home countries and we, I don’t know…if they each may have some little underlying, what they experienced in their home country is different than what they experience here.”
Less forthcoming around police^a^	“…we get called for the eval part, the medical part, but sometimes we have to say, ‘Yeah, I’m here to make sure you’re okay. We’re not with them [the police]’ and then they tell us.”
Provider bias	
EMS feels uncomfortable on scene	“…typical kind of call is going into a [ethnic group] residence, where always, I’m always a little bit on edge…I don’t know how they’re gonna act....I may be profiling them a little bit, but, but, they come from places of violence.…I sometimes have a sense of maybe they don’t trust us, and I think, if you’re in somebody’s house that doesn’t trust you, you always have to be on guard.”
Perception of heightened emotions	“I hate to stereotype or anything, but, you see trends.…Some cultures are very kind of, over-reactant, or some are more stoic and don’t react, you know, so it’s this big spectrum, but this particular instance was the overreact, and ’oh my goodness‘ and everything’s crazy.”

Abbreviations: ALS, advanced life support; EMS, emergency medical services; MI, myocardial infarction.

^a^ Corollary facilitator in Table 3.

^b^ Expansion of theme in eTable 1 in Supplement 1.

^c^ Expansion of theme in eTable 2 in Supplement 1.

Cultural differences were the second most commonly cited barrier. Examples included specific challenges for male providers caring for female Muslim patients while maintaining appropriate cultural modesty and boundaries (Table 2). Female providers are a minority group in EMS^21,22^ and were frequently unavailable. Providers commonly perceived that patients with LEP delayed activating EMS. They cited potential cultural reasons for this based on their observations, including trying traditional treatments for illnesses first and calling friends and family before 911 (eTable 2 in Supplement 1). Providers also perceived that perhaps because of delays, patients with LEP were often more severely ill when they arrived on scene than patients who had English proficiency. While increased illness severity in any individual patient could make their initial on-scene evaluation more straightforward (as noted above), they described this overall trend as a barrier to optimal care for patients with LEP.

High-stress scenarios (eg, shootings, stabbings, and police presence) were also barriers to optimal prehospital care. Providers acknowledged that violent events were stressful regardless of patient and bystander LEP status due to increased bystander tensions and reluctance to give details about the event, but challenging communication due to LEP increased the difficulty of this environment. Providers also perceived that patients with LEP were more likely to have bystander crowding at the scene than English-speaking White patients (Table 2 and eTable 2 in Supplement 1).

Bias and distrust were also described (although not always named or recognized) as barriers to care, frequently conflated with barriers such as cultural differences and high-stress scenarios. Several experiences described involved racial and ethnic minority individuals rather than specifically patients with LEP. Some providers acknowledged feeling wary on scene due to perceptions that patients or bystanders “come from places of violence” and that some cultures are “over-reactant” or “hysteric,” indicating issues with provider profiling, bias, and racism (Table 2). Providers perceived distrust of EMS and law enforcement from some patients with LEP and from individuals from racial and ethnic minority groups and noted that patients were less forthcoming around police. Distrust was sometimes attributed to poor prior experiences with EMS and/or police in the US or elsewhere and was also perceived as a reason for delays in seeking care.

### Facilitators for Optimal Prehospital Care

An on-scene interpreter (bystander or child) was unanimously cited as an important facilitator for providing care (Table 3). If no on-scene interpreter was available, many participants preferred telephone apps over telephonic interpreting (eTable 1 in Supplement 1).

**Table 3.** Facilitators and Strategies for Prehospital Care

Facilitators	Quotes
Interpreter on scene^a^^,^^b^	“…there’s a time component, too, language line seems a little clunky….I will be quicker and get a better result finding somebody [on scene].”
Simplifying speech	“I ask simple...simple questions. Direct, yes or no’s, as simple as I can make it, yeah.”
Severe or unstable patient condition^a^	“You get somebody stabbed in the gut or stabbed in the chest and they don’t speak English, you treat ‘em the same way you do anyone…you do what you need to do, you know? Get some lines, flutter valve ‘em, tube ‘em, what, you know, all that stuff, it doesn’t matter what language they speak.”
Obtain only essential patient history on scene for those with high-acuity disease	“Sometimes [EMS providers] use a language line…but by the time they call ‘em, get the right language, that patient could be in an ambulance to the hospital.”
Rely on objective clinical findings	“…apparently told the dispatch that he’s got chest pain. And you can’t communicate really. Maybe there’s a family member that can help, but essentially you can’t do your comprehensive, thorough, cardiac chest pain, you know, history….So in those cases, we rely, sort of, on our tools, we take vital signs, we do 12 lead EKG, we look at those….We sort of rely on our exam.”
Building trust and rapport^a^	“…family members…they all want to be active and doing something….So it’s actually been really helpful if my driver goes over, talks to mom. My captain goes over, talks to the dad…that helps with a lot of these cultures…they absolutely will fall in love with us and treat us completely differently as soon as we engage all of them, instead of just staring at them because they can’t speak our language.”
Positive prior interactions between EMS and community^a^	“The firefighters…yeah, they have a huge interaction.…They kinda know what’s going on in their communities and are way more tied in [than medics].”
EMS feels welcome on scene^a^	“…most people realize that like, we’re doing the best we can, we’re trying to help them…I’ve never had a problem where I’ve had like an immigrant or somebody who doesn’t speak English be mad at us…I’ve had like a very positive—they get it, they don’t speak English, we don’t speak [other languages]. They’re, you know, trying to get the message to us and we’re trying to get them to the hospital.”
EMS adaptability	“…generally speaking, as much as we can, you try to respect what you can respect and still treat around it. Cuz they were like, ‘Oh my god they have a big deal, like you’re not supposed to touch their head,’ and I used to not know that…if someone’s there to tell you that, and you can work around those things that’s fine.”
Nonverbal communication	“…try to reassure them, with body language. Empathy. You know, being able to convey to them, not necessarily speaking, but conveying to them that everything’s going to be okay, or that you’re doing everything you can to help their loved one.”
Conservative treatment and transport decisions	“I’m a bit more conservative when it comes to [patients with LEP] because I can’t rule things out, and that’s kinda how I practice my medicine anyway…if I can’t rule it out, then, you know…then they’re going [to the hospital] with medics.”
Extra time and resources for interpretation on scene for low-acuity and/or unclear disease presentations	Medic 2: “All comers get the same level of care, period.…In fact, I would say we’re probably more attentive to those individuals, because we’re having such a harder time getting.” Medic 1: “To make sure we aren’t missing something!” Medic 2: “Yeah, exactly!” Medic 1: “Yeah, we probably spend more time than we would [otherwise].”
Police presence for crowd control^a^	“…there have been occasions where we’ve called for police. You know, for sort of crowd control on cardiac arrests. Because, you know, walk in, and there’s a very- there’s a cardiac arrest that we need to work, and there’s family members that are, you know, losing their minds in hysteria, to the point of, you know, needing them to be separated from the scene for us to be able to do our job.”

Abbreviations: EKG, electrocardiography; EMS, emergency medical services; LEP, limited English proficiency.

^a^ Corollary barrier in Table 2.

^b^ Expansion of theme in eTable 1 in Supplement 1.

Emergency medical services providers also universally agreed that high-acuity or unstable patient conditions were a facilitator to providing care as objective findings (ie, hemorrhage, hypotension, and dyspnea) indicated immediate interventions and transport to the hospital with minimal communication. They noted that there were more resources at the hospital to obtain the patient’s history and that their primary concern was getting sick patients to the hospital fast. Providers stated that they relied heavily on objective clinical findings (eg, vital signs, physical examination, and electrocardiography) anytime there was a language barrier with patients.

Building trust and rapport was also described as an important facilitator for interpersonal interactions and providing optimal care. Providers described treating people with respect, nonverbally expressing empathy, respecting cultural practices as much as possible, and involving family in the care of the patient. These strategies required EMS adaptability, another facilitator for optimal care. An important part of adaptability was described as finding a balance between providing care and respecting culture for each situation (Table 3). Preexisting rapport due to prior interactions between local EMS and communities was also described as a facilitator (Table 3). One group of EMS providers described police presence as a facilitator when crowd control was needed.

### Patient LEP Status and EMS Provider Decision-making

As described above, EMS providers stated that their decision-making regarding the care and disposition of unstable or critically ill patients was unaffected by LEP because such decisions were more dependent on objective data and basic examination findings than history or nuanced examination. In contrast, they described spending extra time and resources on scene with patients who had low-acuity concerns or problems of unclear severity. This typically involved using telephonic interpreting to obtain a detailed history (eg, characteristics of abdominal pain, nuanced abdominal examination) and to provide reassurance and instructions for patients who did not need to go to the hospital (Table 3). However, providers acknowledged that they frequently transported patients with LEP to hospitals regardless of perceived illness severity due to concern for miscommunication of symptoms, unrecognized problems, and perceived patient expectations of transport (Table 3). The Figure shows a conceptual model developed from our data outlining the EMS decision-making process and how providers perceived it was impacted by patient LEP status and cultural differences.

**Figure.**
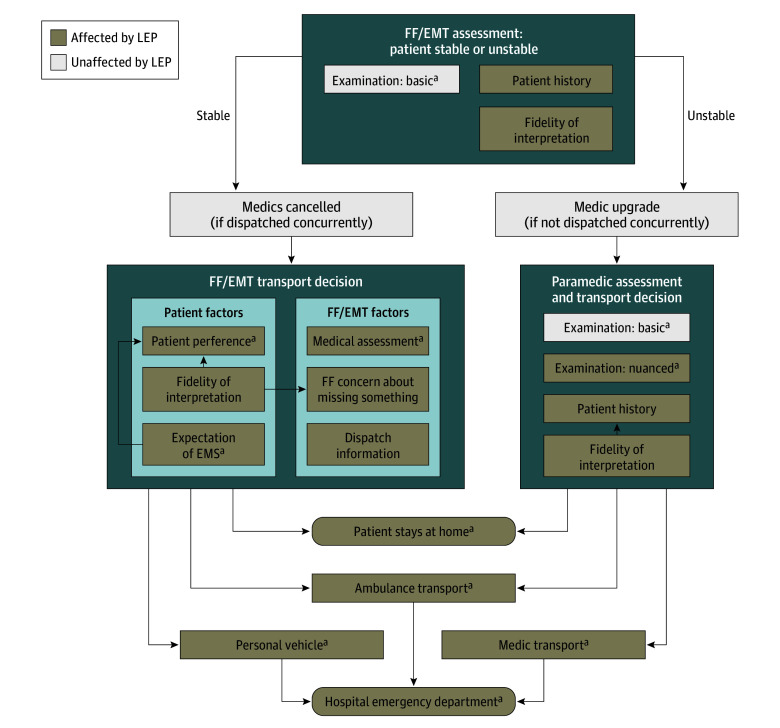
Limited English Proficiency (LEP) and Emergency Medical Services (EMS) Provider Decision-making EMT indicates emergency medical technician; FF, firefighter. ^a^Affected by cultural differences.

### EMS Provider Recommendations for Improvement

Improved speed and technology for language interpretation was the most prominent idea for improving prehospital care for patients with LEP. Education for communities and EMS providers as well as community-EMS interactions outside emergencies to build trust and relationships were other commonly cited strategies for improvement (Table 4). Focused education on local cultural norms and practices was desired by providers, although they did not think general training on the details of multiple cultures would be helpful. Providers also perceived that education on the roles of different EMS providers within the health care system might be helpful for patients with LEP.

**Table 4.** Recommendations for Improvement

Recommendations	Quote
Focused cultural and community education for EMS	FF4: “A list of [cultural] do’s and do nots would be helpful I think.” FF4: “I would also caveat on that, or jump on that as well, a couple, I think, hellos and goodbyes in their native languages.” FF5: “Basic, yeah.” FF4: “You know, I try to pick up on those, it goes a long way for first impression, and calming them down…and also be very beneficial I think to have some kind of cultural awareness of what’s going on.”
Education about EMS for individuals with LEP	“…one of the components that would be great is to have an outreach to the communities, so they understand what they can expect from the first responders that come to them. And I think that crosses over between fire, police, and medic, it doesn’t matter, all of us, of what services we bring, what information they need to have available to us.”
Community-EMS interactions outside emergency situations	“I think [Touch-a-Truck community outreach events] are useful for showing the public we’re here, you know. Making a presence known, and then, obviously the interactions that we have, I would say ninety-nine-point nine percent of the time are positive, between the firefighter, and the mom and her kid, and ‘Oh, that was fun’…so it’s a very positive experience for them, and the firefighters.”^a^
Diversification of EMS workforce	“…in time, our workforces will become more representative of the communities we serve…eventually they’ll integrate into our workforces and be represented in our workforces, and hopefully, they’ll maintain their native tongues.”
Improved speed and technology for interpretation	FF1: “…if dispatch pulled up the appropriate dialect…and then assigned a translator, and then that translator called me?” FF2: “That’d be awesome. Yeah.” FF1: “That would be—I would be more inclined to use it.” FF4: “[Live video interpreter] would need to be like, bring up the tablet, it goes to that select language screen every time, I push the language that they’re speaking and a few rings and someone pops up, you know, ‘Hola!’ ‘Hey man, we got someone that speaks Spanish, we need you to talk’ and it starts right there. And then that’s, and it’s going, and it’s seamless.” Interviewer: “But anything less than that?” FF4: “It would be in the, in the rig.”

Abbreviations: EMS, emergency medical services; FF, firefighter; LEP, limited English proficiency.

^a^ Touch-a-Truck is an event where firefighters take a fire truck to a public place, such as schools, to interact with the community.

Diversification of the EMS workforce was recognized as an important area for improvement, specifically cited as a strategy to improve communication on scene and to improve EMS cultural awareness. Some providers discussed that learning a limited number of key words in different languages such as “hello,” “goodbye,” and “pain” might also help build relationships and improve interpersonal interactions. Many specifically commented that they once had flip charts with pictures and phrases in a few different languages, but utility was limited due to the numerous languages that they encountered.

## Discussion

There are known disparities in prehospital outcomes among patients with LEP,^1,2,3,4,5,6,7,8,9^ and in this qualitative study, EMS providers reported their experiences responding to emergencies for patients with LEP. Participants described many barriers to providing standard care during prehospital emergency response for patients with LEP yet were unaware that these barriers impacted quality of care. The major barriers to optimal care identified were ineffective interpretation, cultural differences, high-stress scenarios (eg, violent events), unclear acuity of a patient’s condition, provider bias, and distrust of EMS. Participants described several facilitators to optimal care, including using an on-scene (bystander) interpreter, relying on objective clinical findings, building trust and rapport, and conservative decision-making regarding treatment and transport. One prior study evaluated strategies used by EMS providers to overcome language barriers,^23^ but to our knowledge, ours is the first study to provide insights into EMS provider perceptions of prehospital care for patients with LEP beyond language barriers.

Although substantial evidence supports that trained, professional interpreters improve care for patients with LEP,^24,25,26^ access to prehospital trained interpreters is limited and connecting to telephonic interpreting is a major barrier for EMS. The time from initial call to connection with the interpreter was the primary delay, and a previous study demonstrated that telephonic interpreting during 911 calls led to increased time to dispatch of paramedics^1,8^ and increased time to initiation of bystander cardiopulmonary resuscitation.^4^ While some providers in our study preferred automated translation via a telephone app to telephonic interpreting, this is a less effective form of interpretation.^27^ Strategies to eliminate connection delays (eg, direct connection via dispatch or video interpreter) may be particularly useful to improve use of certified interpreters.

Despite describing substantial delays with interpretation, EMS providers in this study recalled no delays in on-scene care for patients with LEP, a perception corroborated by studies in New Mexico and Minnesota that reported similar or decreased on-scene times for patients with LEP.^28,29^ Decreased on-scene time may be due to limiting interpreter use and obtaining only essential history for patients with LEP. While this strategy is appropriate for unstable patients, our results suggest that EMS providers may transport more patients with LEP than medically necessary. Improving options for trained interpreters may increase costs initially, but a decrease in costs may ultimately be realized by reducing unnecessary hospital transport and emergency department utilization while facilitating timely, appropriate, high-quality care for patients with LEP.^24,25,26^

While our study did not include patient perspectives, prior studies evaluating prehospital response from the perspective of individuals with LEP substantiated EMS perceptions that patients with LEP may delay or avoid activating EMS.^6,16,17^ In a qualitative study with Chinese-speaking individuals in Washington, participants described negative perceptions of EMS, confusion with the EMS system, concerns about cost, and calling family first as barriers to calling 911.^16^ Latinx individuals in Colorado reported fear of getting involved with the law or litigation, fear of retaliation after reporting a violent event, immigration status, and concerns about cost as reasons to avoid calling EMS.^17^ A survey of Spanish-, Arabic-, and English-speaking caregivers of pediatric patients at an emergency department in Michigan showed that 16% of caregivers with LEP compared with 58% of native English speakers used EMS to get to the hospital.^6^ Concerns about cost, effects on immigration status, inability to communicate with 911 dispatchers, and lack of knowledge of EMS were cited as reasons for not accessing prehospital care.^6^

Another related issue implicated in this study was that EMS providers perceived that many LEP and racial and ethnic minority communities distrusted EMS more when police were present. Distrust and fear of police is a recognized issue and may have worsened since these data were collected in 2018 as social movements,^30^ published academic data,^31,32^ and news media coverage^33,34^ have increased awareness of fatal US police shootings of racial and ethnic minority individuals. Emergency medical services and police response are often intertwined, such as in response to firearm injuries (which have increased during the COVID-19 pandemic^35^), which has likely caused additional strain during prehospital interactions since our data were collected in 2018. This recognized distrust of police also highlights that calling police for crowd control (cited as facilitator) (Table 3) should not be taken lightly.

Some EMS providers acknowledged feeling uncomfortable during interactions with patients from different cultures due to perceived tendencies of violence, indicating bias and racism (Table 2 and eTable 2 in Supplement 1). In our study, perceived cultural differences were a barrier to optimal prehospital response, although racism and bias were often commingled with comments regarding challenges with cultural differences and LEP and were sometimes difficult to separate. Still, our findings suggest bidirectional interpersonal barriers to optimal prehospital care that are modifiable.

The EMS workforce is also mostly composed of White males,^21,22,36^ and diversification would improve prehospital care through improved communication, trust, and interpersonal interactions.^37^ Participants also suggested EMS-community interactions outside emergencies to build trust, cultural familiarity, and relationships. As a specific example, findings from this study informed development of the Working for Equity: WE Stop the Bleed program.^38,39^

Perhaps the most important finding from our study is that EMS participants described many differences in how they provided care for patients with LEP but explicitly expressed a belief that they provided the same high-quality care to all comers regardless of language, race, or ethnicity. There was a lack of recognition of lower quality of care, particularly in the setting of known disparities. Providers consciously desired to do their best for every patient and often worked in suboptimal, high-stress environments. Still, like other health care settings, both individual (eg, preference for noncertified interpreters, subconscious bias) and system factors (eg, lack of integrated interpretation services, lack of workforce diversity, prevalent stereotypes, and systemic racism)^40^ contributed to the lower quality of care described in this study. While the underlying causes for these problems are multifactorial and complex, the first step in working toward equitable high-quality care is recognition that this problem exists.

### Limitations

This study has limitations. The experiences described in this study may not represent EMS interactions with patients from every language and culture and do not provide the patient perspective. Additionally, many of the scenarios discussed involved caring for racial and ethnic minority patients, not exclusively patients with LEP, and thus not all results are specific to our original research question. We specified this in our results and discussion where possible, and still many findings merged race, ethnicity, culture, and language as these were often difficult to isolate. Our results may also have been influenced by social desirability bias—a tendency toward socially acceptable responses—due to sensitive topics of conflict or discomfort with other cultures and suboptimal patient care. We attempted to mitigate this by choosing a moderator with similar age, sex, and race to most participants and professional credibility (EMT-trained) with minimal researcher-participant power differential. Furthermore, there may have been bias due to power imbalance among participants of different ranks. The moderator (D.S.) perceived that the groups generally spoke openly, but some participants may have withheld experiences or opinions that they did not want to share with colleagues of different ranks. We believe that the benefits of participants being among their colleagues for discussion outweighed the potential bias.

## Conclusions

This qualitative study found that EMS providers faced many challenges during prehospital emergency response for patients with LEP that may have led to lower quality of care and unnecessary high rates of hospital transport. Our findings support prior work that improved speed and technology for interpretation are needed but also suggests that cultural differences, provider bias, and distrust of EMS are important considerations. Future work should focus on direct studies with LEP and racial and ethnic minority communities and targeted interventions to improve modifiable barriers such as interpretation, lack of workforce diversity, provider bias, and distrust of EMS. Community education on the roles of EMS providers and law enforcement during prehospital response, ongoing cultural humility training for EMS providers, explicit discussion of quality-of-care issues, and interactions outside emergencies may be of particular importance.

## Notes

Supplement 1. **eTable 1.** Barriers and Facilitators to Effective Interpretation During Pre-Hospital Care **eTable 2.** Emergency Medical Services (EMS) Provider-Perceived Causes for Delay in Activating EMS and Distrust **eAppendix.** Medic/EMT Moderator Guide

Supplement 2.
Data Sharing Statement
