# Supplementary material for: Perceptions of Prehospital Care for Patients With Limited English Proficiency Among Emergency Medical Technicians and Paramedics
Source: JAMA Netw Open. 2023 Jan 27;6(1):e2253364. doi: 10.1001/jamanetworkopen.2022.53364 (PMC11875121; doi:10.1001/jamanetworkopen.2022.53364)
Supplement: Supplement 1. — eTable 1. Barriers and Facilitators to Effective Interpretation During Pre-Hospital Care eTable 2. Emergency Medical Services (EMS) Provider-Perceived Causes for Delay in Activating EMS and Distrust eAppendix. Medic/EMT Moderator Guide [file jamanetwopen-e2253364-s001.pdf]

Supplemental Online Content

Stadeli KM, Sonett D, Conrick KM, et al. Perceptions of prehospital care for patients with limited English proficiency among emergency medical technicians and paramedics. *JAMA Netw Open.* 2023;6(1):e2253364. doi:10.1001/jamanetworkopen.2022.53364

eTable 1. Barriers and Facilitators to Effective Interpretation During Pre-Hospital Care

eTable 2. Emergency Medical Services (EMS) Provider-Perceived Causes for Delay in Activating EMS and Distrust

eAppendix. Medic/EMT Moderator Guide

This supplemental material has been provided by the authors to give readers additional information about their work.

© 2023 Stadeli KM et al. *JAMA Network Open.*

eTable 1. Barriers and Facilitators to Effective Interpretation During Pre-Hospital Care

Barriers	Quote
Inefficient interpretation	*“I used language line a lot at my last department […] but it’s not practical for a lot of what we do […] it takes a while to get connected, sometimes you don’t get the language that you need because the one person who speaks it is already busy”*
Loss of detail and nuance	*“sometimes we’re using 5-year-olds that speak good English, but the word base is so limited for them you have to be, talk in a lot simpler terms”*
Fidelity of interpretation	*“lotta our words don’t cross over […] so they take, creatively, come up with the right word, and is it truly what we’re trying to ask? Because, you know, they’re taking their license […] we say a word and there’s no translation, and then, do they understand our word, and does it get translated right, and does the patient understand it, then, does it get translated back properly.”*
**Facilitators**	
Family/friend interpreter available	*“what ends up happening, is, if there’s school age children there, they’re bilingual. They, they, they know how to speak English. And so we’ll often times use the children as an interpreter, and I think that’s, I would say that’s usually how we kinda figure out”*
Phone-based app translation (Google Translate)	*“…you can say ‘English to Spanish, record voice’ and so I just sort of gave a little report […] Boop! [mimes pressing button] and then [the phone] speaks Spanish to the mom, and then mom was like [mimes sigh of relief][…] it’s very easy, as long as I have cell service, and it does a pretty darn good job at translating.”*
**Either Barrier or Facilitator**	
Remote interpreter	*“…very few times, we actually have to call the language line and try and, and track down an interpreter. That’s very cumbersome and not real user friendly […] but, you know, it’s better than nothing”*

eTable 2. Emergency Medical Services (EMS) Provider-Perceived Causes for Delay in Activating EMS and Distrust

Perceived Cause	Quote
Traditional response to illness first	*“by the time we get there, they’re really sick. Not always, but, we’ve been on some really sick, cuz they tried to treat ‘em with cupping for a couple days.”*
Distrust and fear of law enforcement<br><br>Distrust of 911/EMS	*“one of the things is that the African-American community will shut down around us a lot. I’ve had one [call] on the freeway where she would just not talk to anyone, and I looked around, and there were 3 police officers. She literally stopped in the middle of the freeway and just would not listen to anyone […] when I looked around we had 8 white males and it was just her…she was resisting and trying to run out into traffic…for the most part, they see us just as badly as police”*
Call family/community first	*“They’ll ask someone in their community before they call 911. Because somebody may have been some kind of healer where they came from. You know, and they’ll call them before they call us.”*
Non-citizen status	*“…if there’s people here illegally in the country, undocumented aliens, for sure, they’re gonna be, probably a little bit resistant to calling, for fear of deportation. Especially in the current political climate.”*
Financial concerns of seeking care	*“…they wanna keep gramma home as long as possible, cuz, number one, they might not have the financial means to send them out…”*

© 2023 Stadeli KM et al. *JAMA Network Open.*

Lack of recognition of seriousness of event	*"…mom is gonna stay with us, because that's what you do in those cultures, you take care of your family, and then you didn't realize they're breathing 60 times a minute for the last two days."*

EMS: Emergency Medical Services

Appendix 1: MEDIC/EMT MODERATOR GUIDE

Consent procedure completed

Introduction

(Tape recorder is turned on)

We began recording this interview to make sure we hear everything you are telling us.

The purpose of this interview is to explore your experiences as a prehospital provider interacting with communities who are limited English proficient. In particular, we are interested in your:

- **Overall impressions** of your interactions with communities with limited English proficiency,
- **Communications** during pre-hospital care with those communities,
- **Barriers and facilitators** to providing care during those interactions, and
- **Recommendations** for improving pre-hospital care for communities with limited English proficiency

Just a few logistics and then we'll get started.

- Please feel free to speak openly and honestly
- There is no "right answer" to any of the questions or one "right experience." We are just here to learn from you.
- This interview will last approximately 1 hour. If you need to use the restroom or take a break, please feel free to do so at any time.

Do you have any questions before we get started?

To start, can we go around the room and everyone tell their name, their location/role, and one fun thing you like to do outside work to get to know each other a little bit.

© 2023 Stadeli KM et al. *JAMA Network Open.*

QUESTIONS/PROMPTS

Overall impressions

1. What does a typical workday look like for you?
    a. *Prompts: average number of responses, percent of responses that require emergency intervention, percent that lead to ED transport*
2. On a typical workday, how many calls do you respond to that involve someone with LEP?
3. Think about the last time you responded to a call that involved LEP community members. What happened?
    a. *Prompts:*
        i. *Was the person who needed emergency intervention LEP? Their family members?*
        ii. *How did you assess their needs?*
        iii. *How did they react/handle this situation*
        iv. *Did they get transported to the hospital?*
            1. *can you describe any delays that you remember?*

Communication

4. How often is there someone there who can help interpret?
    a. *Prompt: Who is that person? (child, parent, community member)*
    b. *Did you think the interpretation was accurate?*
    c. *Were there any conflicts that you recall that may have resulted from difficulties in interpretation?*
5. Have you ever had to respond to a call where there was no one to assist in interpreting?
    a. *Prompt: How did you handle that situation?*
6. Beyond language barriers, do you notice different interactions with LEP communities compared to those that are English-proficient?
    a. What do these differences look like?
7. What patterns have you noticed between different LEP communities in the way they interact with EMS?
    a. *Prompt: Women vs. men, different neighborhoods, different ethnicities*

Barriers/facilitators

8. Have you noticed any differences (or problems?) when the patient or family had LEP (in general)? Tell us about a specific time.
    a. *Prompt: BLS/ALS upgrade/downgrade, change in care, change in transport, change in pt outcome*
9. What do you see as the major barriers to you being able to provide services to communities with LEP?
10. What do you see as the major barriers to communities with LEP feeling comfortable with you or giving you the information that you need?
11. Are there any particular health conditions that make communicating more difficult with LEP community members?
    a. *Prompt: Mental health, respiratory distress, cardiac arrest, infants, etc.*
12. In your experience, is there anything that makes communication and interactions between LEP community members and EMS better or easier?

Recommendations

13. What have you found to be the most effective strategies for communicating with LEP communities?
    a. Would you rate one of these as the single most effective strategy overall? Which one?
14. What kinds of approaches do you tend to avoid or would be concerned about using with LEP communities?
15. Are there any strategies that you think would be effective, but are unable to implement for some reason?
16. Are there any prior projects to improve interfacing with EMS that you've been involved with or heard about?
    a. A previous survey of King County fire personnel identified effectively interfacing with LEP patients as one of the greatest perceived needs for training. Can you tell me what that training might look like?

Conclusions

© 2023 Stadeli KM et al. *JAMA Network Open.*

17. Are there any topics/concerns/issues that we did not discuss that you think are important to consider as part of our ongoing efforts to promote communication between LEP communities and EMS?

© 2023 Stadeli KM et al. *JAMA Network Open.*
