# Supplementary material for: Perceptions of Prehospital Care for Patients With Limited English Proficiency Among Emergency Medical Technicians and Paramedics
Source: JAMA Netw Open. 2023 Jan 27;6(1):e2253364. doi: 10.1001/jamanetworkopen.2022.53364 (PMC11875121; doi:10.1001/jamanetworkopen.2022.53364)
Supplement: Supplement 2. — Data Sharing Statement [file jamanetwopen-e2253364-s002.pdf]

Data Sharing Statement

Stadeli. Perceptions of Prehospital Care for Patients With Limited English Proficiency Among Emergency Medical Technicians and Paramedics. *JAMA Netw Open*. Published January 27, 2023. doi:10.1001/jamanetworkopen.2022.53364

Data
Data available: No
